# Hippocampal Physiology, Structure and Function and the Neuroscience of Schizophrenia: A Unified Account of Declarative Memory Deficits, Working Memory Deficits and Schizophrenic Symptoms

**DOI:** 10.3390/bs3020298

**Published:** 2013-06-21

**Authors:** Cynthia G. Wible

**Affiliations:** Harvard Medical School, VA Boston Healthcare System, 940 Belmont Street Psychiatry 116A Brockton, MA 02301, USA; E-Mail: cindy@bwh.harvard.edu

**Keywords:** hippocampus, memory, schizophrenia, parietal, superior temporal sulcus, social cognition

## Abstract

Memory impairment is a consistent feature of the schizophrenic syndrome. Hippocampal dysfunction has also been consistently demonstrated. This review will discuss neurophysiological and neuroanatomical aspects of memory formation and how they relate to memory impairment in schizophrenia. An understanding of the cellular physiology and connectivity of the hippocampus with other regions can also aid in understanding the relationship between schizophrenic declarative or relational memory deficits, working memory deficits and the clinical symptoms of the syndrome.

## 1. Introduction

Declarative memory and working memory deficits have been consistently demonstrated to be associated with the schizophrenic syndrome [1,2]. Although individuals with hippocampal damage can rehearse information across small time intervals and retain it, under normal circumstances, the hippocampus is used to process and integrate information across very brief time intervals; and is active and used during working memory tasks [3,4,5,6]. In fact, many of the hippocampal-dependent memory tasks used with animals in conjunction with hippocampal single neuronal recordings and lesion studies could be classified as working memory tasks (e.g., T-maze, radial arm maze, *etc.*; reviewed in a later section). Schizophrenia is most likely a disorder involving aberrant activation. For this reason, it is important to understand that the role of the hippocampus in memory depends on its functional and structural connection with cortex and, especially, with higher order perceptual areas, where memory is eventually stored. An understanding of the flow of activation and the nature of hippocampal function can illuminate the relationship between perception, misperception (hallucinations and delusions), memory and working memory. In this review, I will discuss the neural basis of declarative, relational and working memory. Evidence for the numerous types of hippocampal abnormality found in schizophrenia will be described [7]. Research will be summarized to show how the cellular physiology, connectivity and neuroanatomy of the hippocampal system may relate to the memory impairment seen in schizophrenia. The review will also present a framework for understanding how the properties of the hippocampal system may relate to other aspects of the schizophrenic syndrome, such as the age of onset and other symptoms of the syndrome [8]. This framework provides for an understanding of the relationship between declarative or relational memory deficits, working memory deficits and other aspects of the schizophrenic syndrome, such as the age of onset and positive and negative symptoms (hallucinations, delusions, attention deficits, flat affect, *etc.*).

## 2. Review—Memory, the Hippocampus and Schizophrenia

Learning and memory are profoundly impaired in schizophrenia, and the impairment is stable over time. A review of 110 studies reported that 101 of those studies found evidence of an impairment in verbal declarative memory [1]. The memory impairment in schizophrenia was found to be selective when compared to other functions, such as abstraction, verbal fluency and motor function [9]. Memory impairments were also shown to be stable and not affected by moderating factors, such as the duration or severity of illness [10]. 

The hippocampus and related structures are essential for the declarative or relational memory function—the type of memory also affected in schizophrenia [6,11]. Correspondingly, abnormalities of the hippocampal system are the most consistently documented morphometric findings in schizophrenia [12,13]. Hippocampal volume reductions have also been confirmed using large numbers of subjects [14] and are convergent with numerous findings of abnormal neuropathology [15]. Hippocampal alterations in functional activity have also been consistently identified in schizophrenia; these will be discussed in more detail below [16]. 

### 2.1. Hippocampal Function, Anatomy and Physiology as It Relates to Schizophrenia

**Figure 1:**
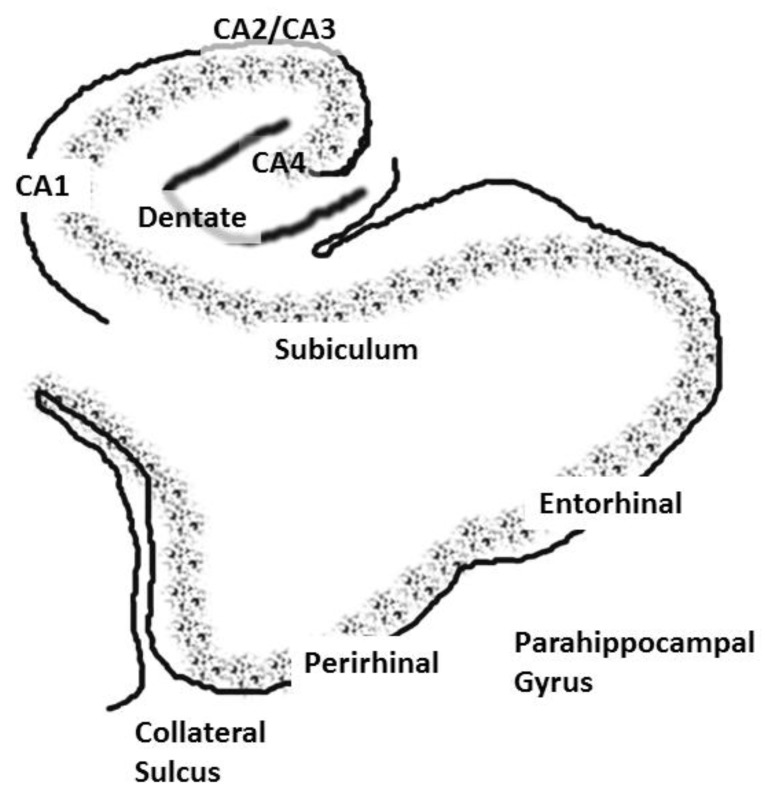
A coronal slice showing the hippocampus and associated structures, including the cornu ammonis, or CA, layers, the dentate gyrus and the subiculum. The entorhinal and perirhinal areas are included in the parahippocampal gyrus.

The role of the hippocampus in relational (declarative) memory is in binding together multiple inputs to create and allow for the storage of representations of the associations among the constituent elements of scenes and events [17]. This function ultimately results in the storage of long-term memory in widespread cortical regions. The hippocampus communicates with widespread regions of cortex through a group of highly interconnected brain regions in the medial temporal lobe (these regions will be collectively referred to as the hippocampal system). Hence, aberrant activation of the hippocampus would affect perceptual cortical regions; especially those showing high functional connectivity with the hippocampal system. The hippocampal system consists of the dentate gyrus, cornu ammonis (CA) fields and the subiculum. The dentate gyrus is an input region, which receives input from the entorhinal cortex. The cornu ammonis (CA) fields of the hippocampus consist of pyramidal cells and are usually subdivided into four regions (CA1–CA4). The area that is often referred to as the parahippocampal gyrus in humans actually consists of several subregions. The dorsal part of the parahippocampal gyrus (inferior to the hippocampal fissure), throughout its extent, is called the subiculum (see Figure 1) [18,19,20]. The entorhinal cortex provides the major input to the hippocampus and also receives output from the CA1 layer via the subiculum [21]. The entorhinal cortex provides input to the hippocampus through two pathways, one projecting to the dentate gyrus and CA3 fields and the other to CA1 and the subiculum. The subiculum then sends a major input back to the entorhinal cortex. The subiculum undergoes an unusual and “striking” increase in myelination during late adolescence; a time when individuals with schizophrenia typically experience the onset of the disease [22]. There is also an increase in hippocampal volume at this time in males, and schizophrenia is more prevalent in males [23].

The entorhinal cortex has its main reciprocal connections with the perirhinal and parahippocampal cortices. Hence, the hippocampus communicates with widespread cortical areas through the entorhinal, perirhinal and parahippocampal cortices [24,25,26]. The hippocampus also contains fiber pathways that run longitudinally throughout its extent (for a discussion, see [27]). This would allow for the excitation between disparate portions of the hippocampal formation. The entorhinal cortex extends from the amygdala (anteriorly) to approximately 10 mm posterior to the most anterior aspect of the hippocampal fissure. The posterior parahippocampal region can be subdivided into cytoarchitechtonic areas, TH (medially) and TF (laterally) according to the nomenclature of von Economo, that extend approximately from the posterior border of the entorhinal cortex to the posterior end of the hippocampus. These regions have a very complex configuration in humans [28]. For this reason, the entorhinal cortex, perirhinal cortex and the TH/TF areas are usually designated as the parahippocampal gyrus and measured together as one region in human morphometric and neuroimaging investigations.

Ischemia and other trauma, such as closed head injury or traumatic brain injury, can produce selective damage to the hippocampus, and this damage is sometimes limited to one or a few subfields [29]. This fact has allowed for advances in understanding the function of the hippocampus. Ischemic damage to even a portion of the hippocampus is sufficient to produce anterograde amnesia for declarative memory and prevents the formation of new declarative or relational memories [30,31,32]. Retrograde memory is memory for information or events that were previously learned. The extent of retrograde memory impairment resulting from hippocampal damage may be dependent on how much of the hippocampal system is damaged. For example, an anterograde memory impairment was evident in patient R.B., who had damage limited to the CA1 layer of the hippocampus, but the impairment was limited to only one or two years [32]. In other words, R.B.’s memory for events up to two years prior to the brain damage were missing, but more remote memories were intact. Patients who had more extensive damage to the hippocampal formation, including all of the CA cell fields, the dentate gyrus and portions of the entorhinal cortex, showed both an anterograde amnesia and a temporally graded retrograde amnesia for 15 to 25 years [30,33]. These findings are important, because they show that the role of the hippocampus is to allow for the consolidation of memory in other cortical regions and that this process proceeds over a number of years. Hence, long-term memory is not actually stored in the hippocampus or, if it is, the hippocampal representation is not necessary for the retrieval of long-term memories after a period of several years. The conceptualization of memory and perception as separate is at odds with this formulation of hippocampal function. Information flows from the cortex to the hippocampus and back out to cortex; this is how memories are formed. In other words, memories are formed via the interplay over time between perceptual regions (including higher order association cortex) and the hippocampus.

The physiology of the hippocampus is unique and endows the region with a high level of plasticity that is important for learning and memory; this property also has important implications for schizophrenia. Neurogenesis also occurs in the hippocampus; hence, it undergoes changes throughout the lifespan [34]. The hippocampus and, in particular, one subfield, the CA1 layer (output layer), has the highest concentration of N-Methyl-D-aspartate (NMDA) receptors in the brain [35]. NMDA receptors are a type of glutamate receptor whose activity underlies long-term potentiation (LTP), a process that may underlie learning and memory [36]. With plasticity comes a propensity for excitotoxic damage; as discussed above, various insults can result in damage that is limited to the hippocampal region. Correspondingly, selective abnormalities of the CA1/subiculum have been shown to be present in prodromal schizophrenic individuals and to be differentially related to the subsequent conversion to psychosis. CA1/subiculum hyperactivity in prodromal individuals uniquely predicted conversion to psychosis and was the only brain region whose activity was correlated with clinical symptoms [37]. Conversion to psychosis was not predicted by activity in the other regions measured, such as amygdala, dorsolateral prefrontal cortex (DLPFC), basal ganglia, gyrus rectus or medial orbitofrontal cortex [37]. 

Hippocampal unit or neuronal activity reflects the fact that higher order representational regions from widespread cortical regions converge within the hippocampal system. Again, since aberrant activation likely underlies at least some phenomenon in schizophrenia, understanding the natural flow of neural activity and the potential consequences of hippocampal over-excitation is important. Single unit (neuronal) activity has been shown to be related to a wide variety of stimuli within various tasks or contexts in humans and other animals, including words, pictures locations, odors and sounds [38,39,40,41,42,43,44,45]. Unit recording studies also show that although hippocampal system function may not be necessary for the maintenance of short-term memory or working memory, it is active during these types of memory tasks [4]. In fact, if the contents of working memory cannot be actively rehearsed or if this process is interrupted, then the hippocampus is needed to “hold” the memory, even at short time periods [5]. Hence, trace conditioning is often affected by hippocampal lesions (where there is a temporal gap between the stimuli used), whereas other types of conditioning are intact [46]. An examination of the connectivity of the hippocampal system along with data from single unit recording in the hippocampus necessitates the view that hippocampus is active during much of daily life. For example, the “place cells” that are recorded in the hippocampus are active regardless of whether or not the memory of spatial location is required at the moment of recording [47,48]; a result also seen for nonspatial stimuli. The online or continuously active role of the hippocampus has recently been formally investigated in recent human neuroimaging studies [3].

The hippocampus may create memory using automatic, obligatory and ongoing binding operations. Relational memory theory [17,38,49] posits that hippocampal-dependent relational processing permits the integration and comparison of discrete experiences and items. In this manner, the hippocampus facilitates the maintenance and integration of the contents of consciousness (consciously perceived stimuli) with representations that are just outside the current contents of consciousness [3]. A relational memory impairment has also been documented recently in schizophrenia [50]. Co-temporal activation of cortical circuits has been shown to be an essential component in the reorganization of cortical representations [51]. The hippocampus may be essential for the association of cortical activation patterns that are in disparate cortical regions or that may be temporally discontiguous [6]. In this way, the hippocampus could allow for the near-simultaneous activation of representations in cortex that were originally processed with a longer time gap between them. This type of simple mechanism could allow for the association of perceptual stimuli with internally activated memories or representations, resulting in the integration of incoming stimuli with existing cortical associative networks. 

The unique physiology of the hippocampus and high concentrations of NMDA receptors allows for relatively high levels of plasticity that are needed for declarative learning and memory. However, as was discussed above, this property also confers a unique vulnerability; NMDA receptor abnormalities have also been proposed to play a major role in schizophrenia and other disorders [52]. In addition to being the most frequent cite of damage after anoxia or ischemia, the hippocampal system (along with the adjacent amygdala) is the most frequent cite of epileptic foci [53]. The sensitivity of the hippocampus to insult may play a role in the development of epilepsy following traumatic brain injury [54,55]. The hippocampus also contains the highest concentration of glucocorticoid (stress hormone) receptors in the brain. These stress hormones can regulate LTP and may increase the likelihood of excitotoxic cell death with prolonged exposure [56,57].

A recent study found that epilepsy and schizophrenia have a familial association [58]. Individuals with a parental history of epilepsy had a two-fold increase in the risk of developing psychosis, compared with those without a parental history of epilepsy. Individuals having a parent with psychosis had a 2.7-fold increase in the risk of having epilepsy. Neurologists have known for some time that patients with temporal lobe epilepsy with a focus in the hippocampus may develop a recurring schizophrenia-like psychosis with delusions or hallucinations [59,60]. The condition can also progress to a longer more chronic psychosis [61]. Activity in the hippocampal system has been recorded using stereoelectroencephalography (SEEG) in patients with schizophrenia-like psychotic symptoms that were related to seizures [62]. These direct brain recordings showed that there was epileptic activity in limbic areas at the time of the psychotic ideation and hallucinations. A recent study reported that a recurrent schizophrenia-like psychosis was actually the first manifestation of what was later found to be epilepsy [63]. Abnormalities of the fast-spiking interneurons that contain the calcium-binding protein parvalbumin have been proposed to underlie schizophrenia (gamma band abnormalities [64]); these same abnormalities have also been implicated in epilepsy [65]. 

These findings provide evidence that the overactivation within the hippocampus may be a cause of the psychotic symptoms that are experienced in schizophrenia [62]. Abnormal hippocampal function, often in the form of overactivation, has also been reported and was linked to schizophrenic symptoms. Abnormally increased activity in the hippocampus and parahippocampal gyrus has been consistently detected [66,67]. This overactivation has been found to precede and to be present during auditory hallucinations and other symptoms [68,69] Schizotypal individuals were found to have an increased duration of activity in the temporal-parietal junction (TPJ) that was highly correlated with symptoms [70].

As an aside, caution is in order when interpreting the data from FMRI studies of abnormal activation in schizophrenia. Whether or not abnormal activity results in a measurement of overactivation or reduced activation depends on the paradigm and types of experimental measures used. For example, in individuals with visual hallucinations (usually due to Charles Bonnet Syndrome), the hallucinations resulted in episodes of increased activation particular visual areas that corresponded to the hallucinatory experience. However, there was also, between hallucinations, a slightly increased baseline of activity in the affected visual regions [71,72]. Hence, neuroimaging tasks that rely on affected regions might show less activity related to the task, because the baseline or comparison condition is abnormally elevated even when the symptoms are not present. Neuroimaging data obtained during episodes of a symptom compared to data obtained when the symptom subsides should show abnormally increased activity that is related to the symptom. This would result in a net decrease in activity during a task condition when the task uses the same system(s) or an increase in resting state measures or connectivity measures. Measuring activity in systems that are normally active both in baseline and task conditions (such as the hippocampus and language regions) can further complicate the interpretation of neuroimaging data, and this must be kept in mind when weighing evidence.

### 2.2. The Interconnectivity of the Hippocampal System: Functional Consequences and Implications for Schizophrenia

**Figure 2:**
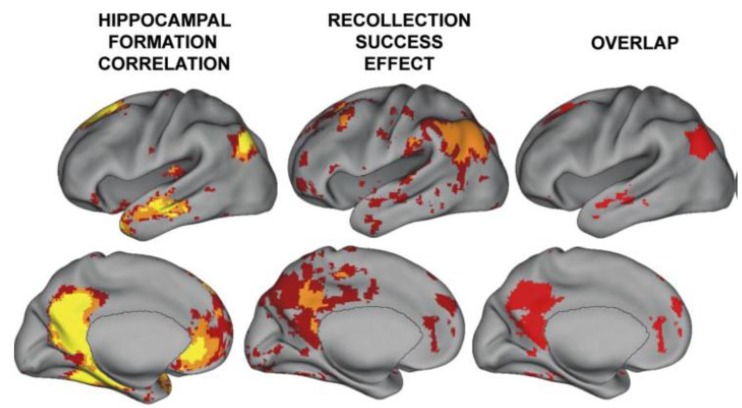
A reproduction of a figure showing regions correlated with hippocampal activity in resting state from reference [74]. These regions overlap with those showing activity related to recollective success. Disclaimer: this is an unofficial adaptation or translation of an article that appeared in a publication of the American Physiological Society. The American Physiological Society has not endorsed the content of this adaptation or translation or the context of its use.

The storage of memory occurs within those regions that are initially used to represent the perceptual or conceptual elements of the memory episode. Overactivation emanating from the hippocampus would activate interconnected cortical regions, resulting in abnormal activity in those regions (but, perhaps, not as severe, long-lasting and with less spread than a seizure). A recent study examined detailed profiles of functional magnetic resonance imaging (FMRI) connectivity within subregions of the hippocampal system using data from 100 subjects [73]. It was found that the temporal-parietal junction (TPJ) was one region showing very high connectivity with the hippocampal system (see Figure 2). Activity in the TPJ predicts recollective success, as well as being a cortical hub and part of what was formerly referred to as the posterior default network [74,75,76]. In addition to being functionally connected, the posterior superior temporal sulcus and inferior parietal regions (part of the TPJ) are highly anatomically connected with the hippocampal system [77,78].

The inferior parietal and posterior superior temporal regions are also implicated in working memory. Lesions of the temporoparietal cortex, or TMS, applied to these regions result in working memory deficits [79,80,81]. Working memory deficits are thought to be a prevalent in schizophrenia and are widely assumed in psychiatry to be related to executive frontal lobe dysfunction and, in particular, to dysfunction of the dorsolateral prefrontal cortex, or DLPFC [2,82,83]. However, as a study performed over two decades ago by Frisk and Milner showed [84], the hypothesized link between working memory maintenance and frontal lobe function is not supported by the evidence [79,85,86,87,88,89,90]. In 1990, Frisk and Milner tested Baddeley’s hypothesis that frontal lobe damage would affect its role as the central executive in working memory and result in a reduced working memory capacity [84]. They did not find evidence for Baddeley’s model of working memory: “Such a relationship was not observed: the number of trials completed correctly on the working memory task as well as the mean span-size of each frontal-lobe group were comparable to those of the other subject groups… ” These findings were replicated in numerous reports, as shown by a careful examination of the literature, where patients with DLPFC lesions were tested in working memory paradigms [91]. It was concluded that none of the studies (representing the performance of 166 individual patients) found that forward verbal or spatial span were impaired as a consequence of frontal lobe damage. The role of the fronto-striatal circuitry in working memory may be in the selection of appropriate responses based on the contents of working memory, not in the maintenance or short-term storage of the items [92,93]. Working memory has been hypothesized to be an emergent property of the recruitment of regions (activity in regions) that are used in perception and representation [86]. Abnormal hippocampal activity coupled with high levels of connectivity between the hippocampus and the TPJ may not only account for some types of memory deficits, but also for other schizophrenic symptoms [8,94,95]. I discussed above how psychotic symptoms were found to stem from abnormal activity (overactivation). One mechanism alluded to above is the direct activation of areas, such as the TPJ by episodic hippocampal overactivation. However, in some cases, the overactivation of a region and, hence, the symptoms, can stem from a lack of input to the region (deafferentation), which subsequently leads to a decrease in the inhibitory tone; this has been well documented in tinnitus, for example; see the discussion in reference [96]. Another example comes from case studies of individuals with epilepsy. In one report, epileptics who underwent a temporal lobectomy (unilateral) and also had a (previously undetected) seizure focus in the remaining hippocampus acquired a psychosis as a result of the temporal lobectomy—and the psychosis evolved over time [97]. In another report, a patient with bilateral circumscribed hippocampal lesions subsequently started experiencing schizophrenia-like auditory verbal hallucinations of a derogatory and commanding nature; the hallucinations appeared concurrently with the patient’s memory deficits [98]. 

As an interesting aside, the patient’s hippocampal lesions were also associated with poor performance on the Wisconsin Card Sorting Test (WCST) [98], which is also assumed to be related to abnormal dorsolateral prefrontal cortex function and has been frequently reported in patients with schizophrenia. This finding replicated an early report of a patient with amnesia; this patient had hippocampal, temporal lobe damage and parietal damage, but the frontal lobes were of normal volume [99]. Although the WCST is often considered to be diagnostic of frontal lobe dysfunction, this assumption is not consistent with the data; see a discussion in the Stefanacci *et al.* (2000) report [99]. For example, a study of 91 patients with focal frontal lobe damage found no consistent relationship between poor WCST performance and frontal damage [100] The Stefanacci and Anderson investigations [99,100] replicated an early study by Teuber reported in 1951, where 131 World War II veterans with brain lesions were studied [101].

There is direct evidence that activity in the human TPJ is correlated with psychosis. The right inferior parietal area was found to be active during delusions [102]. A magnetoencephalography (MEG) study reported that two patients with temporal lobe epilepsy were shown to have “spikey activity” in the right inferior parietal region, concurrent with a delusional state. The activity disappeared when the delusions resolved. 

### 2.3. Hippocampal—TPJ Interaction and the Symptoms of Schizophrenia

The interaction between the hippocampal system and the TPJ may account for the symptoms of schizophrenia. A framework has been proposed to account for the relationship between hippocampal abnormalities and the cognitive deficits and symptoms of schizophrenia [8,94,95]. This account proposes that excitotoxic overactivation of the hippocampus (and possibly the magnocellular system) may excite the TPJ resulting in memory deficits and symptoms. I will briefly summarize this framework here, but see Wible (2012) [8] for a detailed discussion. The TPJ and, in particular, the posterior superior temporal sulcus, plays a key role in the perception of and reaction to dynamic social and emotional gestures, including prosody. This area is also involved in bottom up attention, social attention and the perception of joint attention and of eye gaze [103,104,105] and is differentially active during live social interaction [105]. The TPJ is a core area for theory of mind or understanding and predicting other’s thoughts and actions [106,107]. This region is primarily involved in gesture representation and for the formation of intentions to act (inferior parietal), with audiovisual or speech representation playing a predominant role [108,109]. The region also contains the neural machinery that is essential for self-representation and for agency (visual perspective taking, body schema, vestibular and proprioceptive senses [110]). Inherent in the neuronal representation of dynamic gestures in the TPJ is the representation of intention, agency and anticipation or social expectancy ([111,112,113]; reviewed in reference [8]). The gesture representations are multimodal in the auditory, tactile and visual domain (reviewed in reference [8]). Hence, the erroneous activation of this region could produce the conscious hallucination of a voice with the feeling of an agent who is producing the voice (audiovisual speech), the hallucination of a touch or a conscious visual hallucination of human action or people. It could also produce the feeling of being in a social situation or of the presence of human action with intention, of being watched (eye-gaze) and followed with accompanying feelings of intent and agency; these experiences are present in many schizophrenic delusions, such as delusions of persecution. Aberrant activation in these regions would be expected to disrupt attention, as well as social and emotional perception and reaction, leading to the negative symptoms of schizophrenia. It has been shown experimentally that stimulation of the human TPJ can cause the feeling of a presence or multiple presences, bizarre tactile hallucinations and other experiences that could be classified as schizophrenic hallucinations and delusions [8,94,95,114,115]. The negative symptoms of schizophrenia and the social cognitive deficits also match this region’s core involvement in social cognition, attention and affective responding [8,95].

The medial parietal and medial prefrontal regions are also heavily interconnected with the hippocampus and have been implicated in social functions in neuroimaging studies; however, there have now been several reports of patients with circumscribed lesions of the medial prefrontal cortex in which the patients do not show theory of mind and social cognitive impairments (reviewed in reference [95]). Ventral medial frontal lobe damage (in addition to orbital damage) has been reported to result in a lack of empathy, euphoria, irresponsibility, a lack of concern for the future, as well as a lack of concern for social rules (in conjunction with the intact knowledge for social rules). This syndrome seems more closely aligned with mania, where the symptoms include euphoria, lack of empathy, impulsiveness and a lack of concern for the consequences of behavior, as well as for social rules. Hence, hippocampal overactivation of the TPJ may produce schizophrenic symptoms, whereas an abnormality of the connected medial prefrontal regions may relate more to the symptoms of bipolar disorder. The hippocampus has been implicated in depression, and patients with right TPJ damage sometimes present with syndromes that are similar to those of psychotic depression. These types of symptoms may be related to the region’s role in self-representation and higher order somatic representation (e.g., Cotard’s Syndrome, reviewed in reference [94]). Hence, there may be some overlap between the neural systems involved in schizophrenia and in bipolar disorder, with the hippocampus being a core region.

## 3. Summary and Conclusions

In conclusion, the hippocampus is vulnerable to a number of insults and is prone to excitotoxic activity. Aberrant activity in the hippocampal system could result in the potentiation and activation of representations in highly interconnected regions, such as the TPJ. This proposed link between TPJ function, working memory and schizophrenic symptoms has been demonstrated experimentally [116]. Hence, schizophrenia and related psychoses could stem from a number of etiologies and either from early damage to the hippocampus and/or from some additional genetic abnormality that differentially affects the hippocampal system [117]. For example, mild levels of ischemia or low level exposure to environmental toxins, such as domoic acid, could produce a mild seizure-like state or the propensity for over-excitation in the hippocampus with subsequent progressive cell damage. As discussed above, the association between temporal lobe epilepsy and psychotic schizophrenia-like symptoms have been long noted in the literature [118]. Schizophrenia and epilepsy are genetically related. Domoic acid exposure has been proposed to play a role in temporal lobe epilepsy [119]. This type of disorder would be more difficult to detect than a frank seizure disorder and, hence, could go unchecked and untreated. Depending on the developmental timing of the exposure or insult, this damage could result in either interference in development (autism) or in the potentiation and erroneous activation (schizophrenia) of the representation of human action in the social, emotion and language domains, as well as memory problems. Abnormal activity shared between the hippocampus and TPJ could result either in a lack of development or in the abnormal processing (respectively) of several functions, such as language (audiovisual speech), emotional and social response, social attention, self-representation, theory of mind and the representation of social action, working memory and declarative or relational memory (to name a few functions).
